# Received Signal Strength Recovery in Green WLAN Indoor Positioning System Using Singular Value Thresholding

**DOI:** 10.3390/s150101292

**Published:** 2015-01-12

**Authors:** Lin Ma, Yubin Xu

**Affiliations:** Harbin Institute of Technology, Communication Research Center, 92 West Dazhi Street, Nan Gang District, Harbin 150001, China; E-Mail: ybxu@hit.edu.cn

**Keywords:** indoor positioning, received signal strength, matrix completion, green WLAN

## Abstract

Green WLAN is a promising technique for accessing future indoor Internet services. It is designed not only for high-speed data communication purposes but also for energy efficiency. The basic strategy of green WLAN is that all the access points are not always powered on, but rather work on-demand. Though powering off idle access points does not affect data communication, a serious asymmetric matching problem will arise in a WLAN indoor positioning system due to the fact the received signal strength (RSS) readings from the available access points are different in their offline and online phases. This asymmetry problem will no doubt invalidate the fingerprint algorithm used to estimate the mobile device location. Therefore, in this paper we propose a green WLAN indoor positioning system, which can recover RSS readings and achieve good localization performance based on singular value thresholding (SVT) theory. By solving the nuclear norm minimization problem, SVT recovers not only the radio map, but also online RSS readings from a sparse matrix by sensing only a fraction of the RSS readings. We have implemented the method in our lab and evaluated its performances. The experimental results indicate the proposed system could recover the RSS readings and achieve good localization performance.

## 1. Introduction

Currently, wireless local area networks (WLANs) based on IEEE 802.11 are widely deployed in the indoor environment for mobile devices to access the internet. The high bandwidth, mobility and reliability provide users with a very convenient and economical data communication solution [1,2]. Thousands of access points are being deployed rapidly, not only in the offices and campuses, but also in airports and shopping malls throughout the world. In the meanwhile, due to its ubiquitous network architecture and no additional hardware requirements, the WLAN indoor positioning system based on received signal strength (RSS) has become the most popular option for indoor localization and navigation, as it offers the merits of relative measurement simplicity and minimal hardware requirements to provide a very beneficial supplement to the WLAN application.

However, recent researches show that access points deployed in WLANs are seldom used at their peak capacity. The majority of the access points are frequently in idle status [3,4]. Therefore, in the wake of WLAN developments, the concept of green WLAN is proposed and analyzed to reduce power consumption while guaranteeing the overall performance of the network at the same time [3,5,6]. On the other hand, IEEE has announced its standard to support power saving in WLANs [7]. Many world class network equipment companies, such as Cisco, are developing their new green product lines to support this work on-demand strategy. These definitely provide strong support for the implementation of green WLANs in the real world [8–10]. The basic idea of a green WLAN is a working on-demand strategy by centrally controlling the access points' working status and powering off those ones which are in idle [11,12]. This strategy will no doubt contribute a lot for energy efficiency but also poses severe challenges for the green WLAN indoor positioning system.

Generally, there are two types of RSS-based localization methods for WLAN indoor positioning systems. One is the radio propagation method, where RSS readings are collected to triangulate the mobile device location [13]. The other one is the fingerprint method, where a database pre-built in the offline phase called radio map is required to compare with the RSS readings collected in the online phase. For the fingerprint method, a popular solution is the use of statistical algorithms. Mobile device location is estimated by analyzing the probability of each location's RSS readings based on Bayesian theory [14,15]. In particular [16,17] proposed a new *n*-gram augmented Bayesian method for room localization. The other one used for the fingerprint method is the decisive algorithm. The location of the mobile device is decided by comparing the Euclidean distances in RSS space, which is often referred as the k nearest neighbor (KNN) algorithm [18]. Though many novel algorithms have been proposed recently as candidates to improve the positioning performance, the most widely adopted one is the KNN algorithm, when the computational complexity and estimation accuracy are taken into consideration [19,20].

However, when the access points in a green WLAN are powered off randomly according to the data communication demands, the KNN algorithm is seriously challenged in both the offline phase and online phase by the working on-demand strategy. The immediate impact is that missing RSS readings from unavailable access points lead to the asymmetric matching for the RSS dimensions. Since the RSS readings stored in the radio map and collected in the online phase differ in dimensions, all the fingerprint algorithms will fail to provide the correct location estimation. Therefore, in this paper we propose to implement the singular value thresholding (SVT) theory to recover the missing RSS readings both in the offline phase and online phase, which could help validate the KNN algorithm to provide localization estimations in green WLANs.

SVT theory is derived from the matrix completion problem, which aims to recover an unknown matrix when only a fraction of its entries are known. Matrix completion is not ill posed if some constraints are satisfied [21]. It has already been applied in sensor networks, emitter tracking, *etc.* [22,23]. In the early stage, matrix completion mainly focused on the Euclidean distance matrix completion problem under the premise that the unknown matrix is simple and symmetric [24]. In the years that followed, several approaches were proposed to solve these problems by expressing it as a non-convex optimization problem, which makes use of the particle positions as variables by a modified Newton or quasi-Newton method [25]. Compared with the methods discussed above, the SVT theory is a novel technique, which is easy to implement and effective in computational cost and storage requirements. It considers the matrix completion as a convex relaxation of a rank minimization problem, and approximates the matrix with minimum nuclear norm among all matrices obeying a set of convex constraints [21]. The detailed tight analysis of the convex relaxation is analyzed and proved in [26]. The SVT theory employs the shrink iteration algorithm by carrying out the singular value decomposition on the unknown matrix. In each iteration step, a soft thresholding operation is performed to the singular value, and then followed by a projection onto the known entries [27,28].

Therefore, in this paper, we propose a green WLAN indoor positioning system using SVT theory. In the offline phase, the RSS readings are preprocessed to eliminate the outliers and additive white Gaussian noise. The radio map is implemented by a shrink iteration algorithm for recovery. In the online phase, we propose to combine the RSS readings with the recovered radio map together for recovery. After shrink iteration, RSS readings are then separated out and ready to estimate mobile device location based on the fingerprint algorithm. We have implemented the proposed indoor positioning system in a typical office environment. The experimental results demonstrate the feasibility and good performance of our proposed method. The remainder of this paper is organized as follows: Section 2 will analyse the WLAN indoor positioning system based on a fingerprint algorithm. Section 3 will provide a detailed discussion on how the proposed method recovers the missing RSS readings in both the offline and online phases. Section 4 will investigate the performances of the proposed system. Finally, the conclusions will be drawn in Section 5.

## 2. WLAN Indoor Positioning System

A typical WLAN indoor positioning system involves an offline phase and an online phase. The main task of the offline phase is to build a radio map, which is a template to bridge RSS readings and known locations. For the online phase, mobile devices collect RSS reading vectors to estimate their locations based on a fingerprint algorithm.

### 2.1. Fingerprint Algorithm

The fingerprint algorithm is one of the RSS-based localization techniques used in WLAN indoor positioning systems. In the offline phase, a database called radio map is pre-built. It includes large numbers of reference points, whose locations and corresponding RSS readings are known. Usually, reference points should be carefully set to describe the indoor electromagnetic environment as precisely as possible. In the online phase, mobile devices hence could estimate their locations by comparing the similarity between the online RSS readings and the RSS readings stored in the radio map.

Supposing there are *m* access points and *n* reference points in the localization area. Let a RSS reading stored in radio map be denoted as {**ψ̄***_ij_* : i=1,…,*m*; *j* = 1,…,*n*} (in dBm scale), and its corresponding location is (*x_j_*, *y_j_*). Apparently, the element (*x_j_*, *y_j_*) could be easily expanded into a 3D scenario if altitude is included, but for the brevity of discussion, we ignore the altitude and merely consider the 2D scenario. In the online phase, a vector of RSS reading **ψ̂** ∈ ℝ;*^m^*^×1^ is collected, and then a comparison is processed for further estimating the mobile device location. According to the KNN algorithm, the estimation is based on the RSS Euclidean distance:

(1) $$\begin{array}{cc} d_{j}={\left\|{\bar{\psi }}_{j}-\hat{\psi }\right\|}_{2} & \forall j=1,\ldots ,n \end{array}$$

where ‖ · ‖_2_ is *l*_2_ norm operator, *d_j_* is the RSS Euclidean distance, and **ψ̄***_j_* is the RSS vector in the radio map, which we will discuss later.

Then *K* (*K* > 1) reference points with the smallest *d_j_* are chosen to estimate the mobile device location (*x̂*, *ŷ*) by averaging the known locations of these reference points (*x_j_*, *y_j_*) as follows:

(2) $$\left(\hat{x},\hat{y}\right)=\frac{1}{K}\sum_{i=1}^{K}\left(x_{i},y_{i}\right)$$

In conclusion, we could see that radio map plays a key role in the WLAN indoor positioning system. It bridges the relation between the locations and RSS readings to assist the mobile device to make a proper location estimation.

### 2.2. Radio Map Overview

As discussed above, we could see that radio map generally contains two pieces of information: the reference point's known location and its corresponding RSS readings. Let **L** ∈ ℝ*^n^*^×1^ be the known locations denoted as:

(3) $$\text{L}=\left[\left(x_{1},y_{1}\right),\left(x_{2},y_{2}\right),\ldots ,\left(x_{n},y_{n}\right)\right]$$

Suppose **ψ̂** ∈ ℝ*^m^*^×^*^n^* to be the RSS part of radio map:

(4) $$\Psi =\left[\begin{array}{llll} {\bar{\psi }}_{11} & {\bar{\psi }}_{12} & \cdots & {\bar{\psi }}_{1n}\\ {\bar{\psi }}_{21} & {\bar{\psi }}_{22} & \cdots & {\bar{\psi }}_{2n}\\ \vdots & \vdots & \vdots & \vdots \\ {\bar{\psi }}_{m1} & {\bar{\psi }}_{m2} & \cdots & {\bar{\psi }}_{mn} \end{array}\right]$$

where **ψ̄***_ij_* is the average of RSS readings from the *i*-th access point at the *j*-th reference point. Actually, each column in Equation (4) is a vector representing RSS readings collected at each reference point as we discussed in Equation (1). It could be denoted as:

(5) $$\begin{array}{cc} {\bar{\psi }}_{j}={\left[{\bar{\psi }}_{1j},{\bar{\psi }}_{2j},\cdots ,{\bar{\psi }}_{mj}\right]}^{\text{T}} & \forall j=1,\cdots ,n \end{array}$$

where (·)**^T^** is the matrix transposition operator. Then radio map could be generally expressed as:

(6) $$\text{RadioMap}={\left[{\text{L}}^{\text{T}},\Psi^{\text{T}}\right]}^{\text{T}}$$

**Figure 1.**
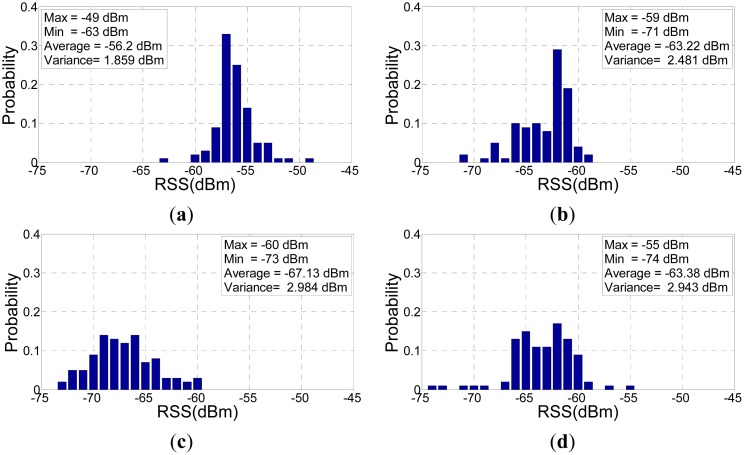
An example of RSS readings collection when mobile device is oriented in different directions (100 samplings for each figure). (**a**) θ = 0°; (**b**) θ = 90°; (**c**) θ = 180°; (**d**) θ = 270°.

Usually, RSS readings stored in the radio map are in terms of average values but not the instantaneous values, which means they are a statistical value obtained over a period of time. This is because the average value could better represent the indoor electromagnetic environment by reducing channel additive white Gaussian noise, and in addition it could provide operation convenience for the online phase. However, care should be taken to eliminate the effects of RSS variations when averaging the RSS readings. Relative orientation of the mobile device antenna towards access point should also be considered due to its significant impact on RSS readings. Suppose $\psi_{ij}^{\theta }$ is a sample of RSS reading, where θ ∈ Θ = {0°, 90°, 180°, 270°}, and the average of the vector is ${\bar{\psi }}_{ij}^{\theta }$. Figure 1 illustrates an example of 100 samples of measured RSS readings.

In order to eliminate the orientation problem, [19] proposed to store these vectors in the radio map. This is a good method to ensure the orientation diversity but will no doubt enlarge the radio map and increase the extra calculation burden according to Equation (1). In order to reduce the computational complexity, we average the RSS readings from all the orientations in this paper.

**Figure 2.**
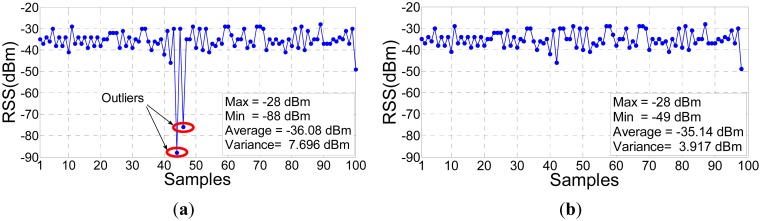
Filtering implementation to eliminate outliers from raw RSS readings (100 samples in the raw RSS readings and 2 of them are eliminated). (**a**) Raw RSS readings; (**b**) Filtered RSS readings.

On the one hand, RSS readings are affected severely by the channel multipath fluctuations, such as pedestrians passing, doors opening and closing, which lead to randomly generated outliers, as shown in Figure 2a. If these outliers are averaged with the raw RSS readings, they will inevitably introduce extra noise into the radio map, and degrade the localization performance in the online phase, so it is necessary to preprocess and diagnose the raw RSS reading to remove these measurement errors. An example is shown in Figure 2b.

There are many algorithms that could filter these outliers out of the raw RSS readings. In this paper we utilize a very practical method by removing these outliers whose RSS are greater than three times the standard deviation of the raw RSS readings. Suppose the standard deviation of RSS readings is $\phi_{ij}^{\theta }$, and any sample of RSS reading $\psi_{ij}^{\theta }$ will be eliminated if the inequality $\left|\psi_{ij}^{\theta }-{\bar{\psi }}_{ij}^{\theta }\right|>3\phi_{ij}^{\theta }$ is satisfied. Therefore, the average of RSS reading stored in the radio map for the *i*-th access point at the *j*-th reference point is defined as:

(7) $$\begin{array}{ll} \psi_{ij}=\sum_{o=1}^{4}{\bar{\psi }}_{ij}^{\theta } & i=1,\cdots ,n;j=1\cdots ,m\\ \text{s}.\text{t}.\left|\psi_{ij}^{\theta }-{\bar{\psi }}_{ij}^{\theta }\right|\leq 3\phi_{ij}^{\theta } & \theta \in \Theta =\left\{0{}^{\circ},90{}^{\circ},180{}^{\circ},270{}^{\circ}\right\} \end{array}$$

In conclusion, once the radio map is built, we could implement the fingerprint algorithm to estimate the localization of the mobile device, but all these processes are under the premise that all access points are powered on. In the next section, we will discuss the green WLAN scenario.

## 3. Matrix Completion in a Green WLAN

The previous section discusses the traditional WLAN indoor positioning system. Now we will investigate the green WLAN scenario. As we stated above, we know the known locations of reference point could be obtained very easily, so that we would focus on the more complex part of the radio map. For the brevity of discussion, we will call the RSS part of the reference points directly as radio map here and in the following discussions.

### 3.1. Samples of RSS Reading Missing

**Figure 3.**
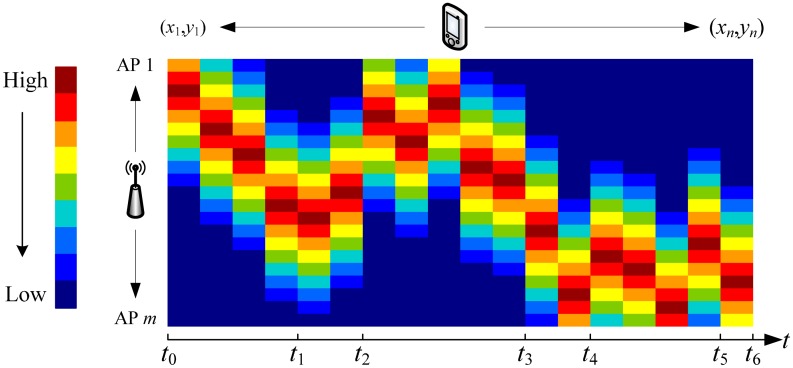
The desired radio map in WLAN positioning system.

It is well known that building a radio map is a very time consuming task. Usually, RSS readings are collected in different periods of time. Figure 3 illustrates a desired radio map built or increased in different times step by step. Here we utilize the graphical representation method to exhibit the radio map, which has the same meaning as the definition in Equation (4). The number of the columns is equal to the number of reference points, and so it has the same number of rows as the number of access points. Each entry value is expressed in colors, where dark red means the RSS reading is very strong, and dark blue means just the reverse. The time bar shows that it takes six periods of time to complete collecting RSS readings for the entire localization area.

Due to the fact mobile diversity will introduce noise into the radio map, we assume the offline and online RSS reading are recorded by the same mobile device in this paper, not only for the brevity of discussion but also for the practice of implementation as what the traditional WLAN indoor positioning system does. It is worth noticing that, compared with the online phase RSS recording, the radio map built in offline phase will significantly affect the experiments results, which we will discuss later.

**Figure 4.**
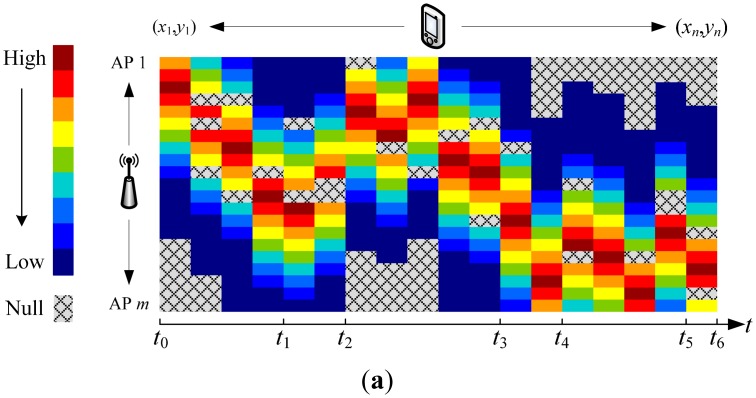

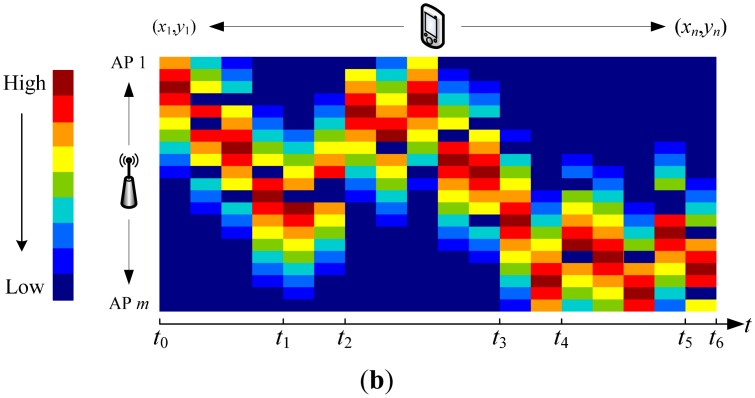
Radio map in green WLAN positioning system. (**a**) Radio map with holes due to no RSS reading sensed; (**b**) Holes are filled arbitrarily with −100 dBm.

Generally, the access point is unavailable for mobile devices when one of the following two cases happens. One is when the signal is blocked by walls and doors, and the other is when the access points are powered off based on the working on-demand strategy, when either of these cases occurs, the mobile device would sense no signal. We show this in Figure 4a, where the grey color means no RSS reading is obtained. We call these entries without value the holes. Conventionally, these holes are filled arbitrarily with a constant value (such as −100 dBm) in order to enable the fingerprint algorithm, where Figure 4b shows the filled radio map. For the online phase, the RSS readings are also implemented in the same way.

Missing RSS readings will directly cause the asymmetric matching problem between the offline phase and online phase, and lead to the failure of the fingerprint algorithm. According to Equation (1), KNN searches through the radio map to get the Euclidean distance between RSS readings online and those stored in the radio map. If any entry is missing, the location of the mobile device will fail to be estimated. It is worth pointing out that we could not ignore these access points with RSS missing readings and use the rest of the available access points to calculate the Euclidean distance. This is because the RSS Euclidean distance comes from different access points, in consideration of the fact RSS readings are missing randomly in the radio map and each of the RSS vectors may have different dimensions as Figure 4a shows. Furthermore, we should know that filling the missing RSS readings with constant values (such as −100 dBm) which happens in the conventional methods will seriously reduce the localization performance, which we will discuss in our implementations result in Section 4.2. In order to guarantee the feasibility of the fingerprint algorithm in the green WLAN, these missing RSS readings should be carefully and properly recovered.

### 3.2. Matrix Completion for Offline Phase

SVT theory is an effective technique for matrix completion. It aims to recover an unknown matrix with a very limited number of entries available under two premises, random observation and low rank. For the former, the matrix completion could not be tolerated with all the entries missing in one column or row. For the latter, the premise of low rank (or approximate low rank) is required so that the matrix can be recovered exactly by solving a simple convex optimization problem [29]. In a green WLAN, the access point working status changes randomly makes RSS readings be missing randomly. In addition, reference points usually outnumber access points in a WLAN indoor positioning system, which makes it true that the radio map is a low rank or approximate low rank matrix. We hence could conclude that both of the constraints are well satisfied in the scenario of concern.

Suppose the desired radio map (without any holes) is **Ψ** ∈ ℝ*^m^*^×^*^n^*, with its entry **ψ̄***_ij_* defined in Equation (7). We sense a fraction of the RSS readings {**ψ̄***_ij_* : (*i*, *j*) ∈ **Ω**} from **Ψ** in the green WLAN, where **Ψ**_sensed_ is a subset of **Ψ** as shown in Figure 4a. Matrix completion by SVT is able to recover **Ψ** from **Ψ**_sensed_ as precisely as possible, and we could model this scenario as a convex relaxation of a rank minimization problem defined as follows:

(8) $$\begin{array}{l} \min{\left\|\Psi_{\text{sensed}}\right\|}_{*}\\ \begin{array}{cc} \text{s}.\text{t}.{\bar{\psi }}_{ij}^{\text{sensed}}={\bar{\psi }}_{ij} & \forall \left(i,j\right)\in \Omega \end{array} \end{array}$$

where ‖ · ‖_*_ is the nuclear norm. And the problem could also be expressed as:

(9) $$\begin{array}{l} \min{\left\|\Psi_{\text{sensed}}\right\|}_{*}\\ \text{s}.\text{t}.\Psi_{\text{sensed}}=P\left(\Psi \right) \end{array}$$

where the projection operator *P*(•)vanishes those entries outside of **Ψ**_sensed_:

(10) $$P\left(\Psi \right)=\{\begin{array}{ll} {\bar{\psi }}_{ij} & \text{if}\left(i,j\right)\in \Omega \\ 0 & \text{if}\left(i,j\right)\notin \Omega \end{array}$$

The SVT theory solves the nuclear norm minimization as stated above in Equation (9) by implementing the shrink iteration:

(11) $$\{\begin{array}{l} \Psi_{\text{sensed}}^{k}=\text{shrink}\left({\text{Y}}^{k-1},\tau \right)\\ {\text{Y}}^{k}={\text{Y}}^{k-1}+\delta P\left(\Psi -\Psi_{\text{sensed}}\right) \end{array}$$

where *k* is the iteration step number.

The iteration step size δ can be any real number but under the condition 0 < δ < 2 for the convergence purpose based on SVT theory. We here set δ = 1.2 *m*/(m − 1). This means the quantity of access points *m* should be more than 3, which is a reasonable assumption in a real WLAN environment. τ is the shrink operator threshold, which is suggested to be set at 5(*mn*)^−1/2^ in [21] according to the iterative Lanczos algorithm. The Lanczos algorithm computes the singular values and singular vectors directly by using the Lanczos bidiagonalization algorithm with partial reorthogonalization.

**Y** is a temporary matrix, which always remains sparse in each iteration in Equation (11) due to $P\left(\Psi -\Psi_{\text{sensed}}\right)=P\left(\Psi \right)-P\left(\Psi_{\text{sensed}}^{k}\right)$. In other word, the iteration for **Y***^k^* only updates those sensed RSS readings {**ψ̄***_ij_* : (*i*, *j*) ∈ **Ω**}, and implements the shrink operation on other unsensed entries {**ψ̄***_ij_* : (*i*, *j*) ∉ **Ω**} for matrix completion by setting a threshold to the singular values:

(12) $$\text{shrink}\left(\text{Y},\tau \right)=\text{U}\underset{i=1,\cdots ,r}{\text{diag}}\left[\max\left(0,\sigma_{i}-\tau \right)\right]\text{V}$$

where **U** and **V** are respectively left and right singular value vectors of **Y**, σ_1_ ≥ σ_2_ ≥ ⋯≥σ*_r_* > 0 are the ordered singular values, diag[·] is a diagonal matrix with the diagonal entries given in the argument of the operator.

The temporary matrix **Y** is initialized by:

(13) $${\text{Y}}^{0}=k_{0}\delta P\left(\Psi_{\text{sensed}}\right)$$

where *k*_0_ is defined as:

(14) $$k_{0}=\left\lceil \frac{\tau }{\delta {\left\|\Psi_{\text{sensed}}\right\|}_{F}}\right\rceil$$

where ‖ · ‖_F_ is the Frobenius norm, and the operator ⌈·⌉rceil; rounds the element to the nearest integer greater than or equal to it.

We can obtain the optimal solution $\Psi_{\text{sensed}}^{k}$ as the recovered radio map until the shrink iteration stops when the following condition is satisfied:

(15) $$\frac{{\left\|P\left(\Psi_{\text{sensed}}^{k}-\Psi \right)\right\|}_{\text{F}}}{{\left\|P\left(\Psi \right)\right\|}_{\text{F}}}<\varepsilon$$

where ε is the tolerance error. We set it as 10^−4^ for shrink iteration stop condition.

In the end of the operation, if **ψ̄***_ij_* is smaller than −100 dBm due to the shrink iteration operation, it is necessary to adjust them as:

(16) $${\bar{\psi }}_{ij}=\{\begin{array}{ll} {\bar{\psi }}_{ij} & \text{if}\;{\bar{\psi }}_{ij}>-100\text{dBm}\\ -100\text{dBm} & \text{if}\;{\bar{\psi }}_{ij}\leq -100\text{dBm} \end{array}$$

### 3.3. Matrix Completion for Online Phase

**Figure 5.**
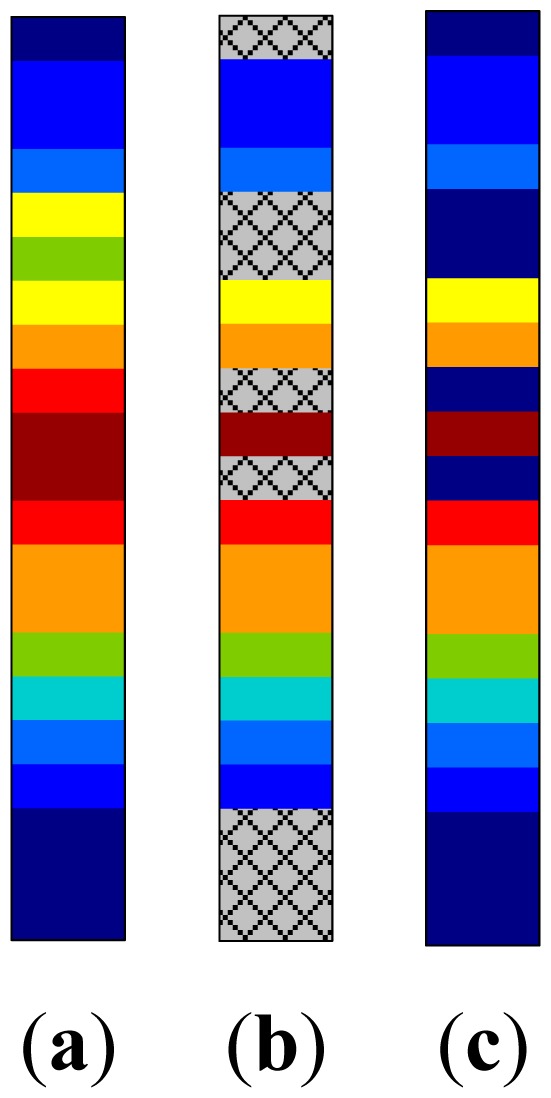
An example of RSS reading collected in green WLAN, where some RSS reading vector missing its entry value in the online phase. (**a**) Ideal RSS vector; (**b**) Sensed RSS vector; (**c**) Recorded RSS vector.

The online RSS readings will also suffer access points are unavailable. As we stated in Section 2.1, the ideal RSS reading in the online phase **ψ̑***_j_* is a vector shown in Figure 5a, when all of the access points are powered on and the mobile device could precisely sense their RSS readings. In this case, the dimension of **ψ̑***_j_* is compatible with the radio map which we obtained in the offline phase. However, in a green WLAN scenario, the actual collected online RSS reading is unfortunately the one shown in Figure 5b, where some of its entries are unavailable and arbitrarily filled with −100 dBm. Figure 5c illustrates the filled online RSS readings.

**Figure 6.**
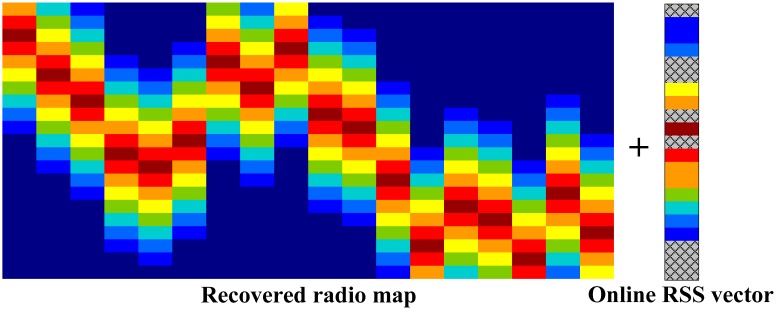
An example of recovering the online RSS reading vector.

This filling process will no doubt degrade the localization performance. In order to recover the online RSS reading, we propose to combine the online vector with the recovered radio map to form a new matrix $\Psi_{\text{sensed}}^{\prime }$ ∈ ℝ;*^m^*^×(^*^n^*^+1)^. Apparently, the online RSS vector could be inserted between any two columns of the radio map. For the brevity of discussion, we assume that the online RSS reading vector is inserted in the last column shown in Figure 6.

Since the new matrix $\Psi_{\text{sensed}}^{\prime }$ is also satisfied with the two constraints, which are random observation and low rank, we could implement the shrink iteration on the new matrix $\Psi_{\text{sensed}}^{\prime }$ for recovery. The calculation flow is the same as we have discussed in Section 3.2. Once the $\Psi_{\text{sensed}}^{\prime }$ is recovered, the last column of $\Psi_{\text{sensed}}^{\prime }$ is the recovered RSS readings for the online phase.

## 4. Implementation and Performance Analysis

So far, we have discussed the SVT theory to make it possible that RSS readings both in the offline phase and online phase are free from the problems caused by the working on-demand strategy in a green WLAN. In this section, we will provide a detailed evaluation of the proposed method.

### 4.1. Experiment Environment

**Figure 7.**
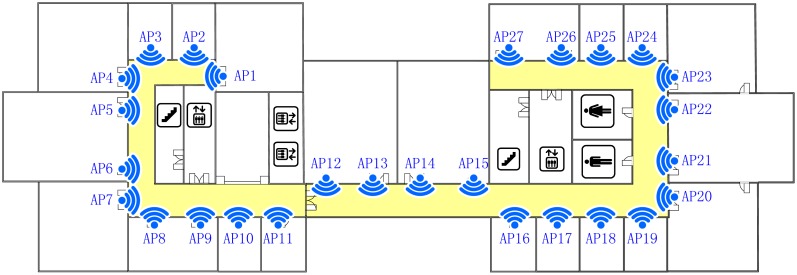
Floor plan for indoor localization, where the area colored in yellow is used for testing.

The experiment environment we build is located in our lab, which is a typical office environment in Building 2A of the Harbin Institute of Technology Science Park. The floor plan for the experiment is shown in Figure 7, In order to provide a signal full coverage, 27 access points (Linksys WRT54G) with IEEE 802.11b/g mode are deployed on top of each room door. The area of interest for localization is the corridor with 49.4 m in length and 14.1 m in width, and it is illustrated with yellow color.

**Figure 8.**
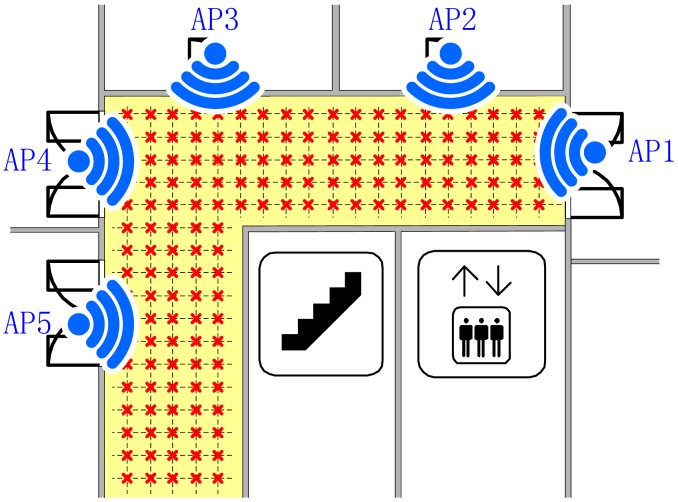
Illustration of reference points distribution in the interesting area for localization.

In our experiment, we divide the corridor into several grids of 0.5 m × 0.5 m, which means the interval between any two adjacent reference points is 0.5 m. There are 823 references points in total, which are marked in Figure 8 with little red crosses. We use a laptop (Lenovo V450) as the mobile device to collect RSS readings both in the offline phase and online phase. At each reference point, we collected 100 samples of RSS reading for each orientation at 2 Hz sampling rate, so that 400 samples of RSS reading per reference point are collected as the raw RSS readings.

**Figure 9.**
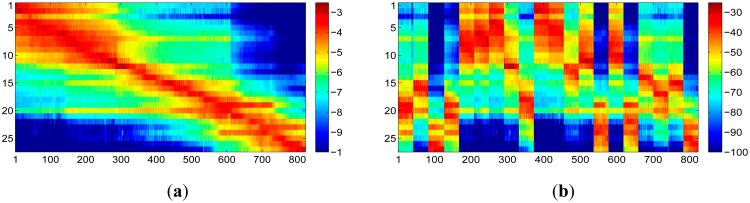
The columns of the radio map are randomly realigned to simulate the scenario that radio map is increased with more interested area for localization requirement. (**a**) Original radio map; (**b**) Realigned radio map.

We first filtered the raw RSS readings based on the discussion in Section 2.2. Then 10 samples from the filtered data in each of the orientation are randomly chosen and averaged as the online RSS readings. This means we have also 823 samples of RSS readings for the online phase. The rest of RSS readings are averaged based on Equation (7) to build the radio map. The actual radio map we got is shown in Figure 9a. The legend bar in colors is in dBm scale. Based on the floor plan, it is easy to tell that the RSS readings are collected anticlockwise step by step, starting from the top left corner and ending in the top right corner. In order to provide a more general case, we suppose radio map is built and increased according to the localization requirements. We assume radio map is built in 20 periods of time, and in each period about 40 adjacent reference points are measured. The columns of radio map are randomly realigned in group of 40 adjacent columns. Figure 9b shows the realigned radio map. From here and in the sequel, all the experiment analysis will base on this assumption.

### 4.2. Radio Map Recovery

**Figure 10.**
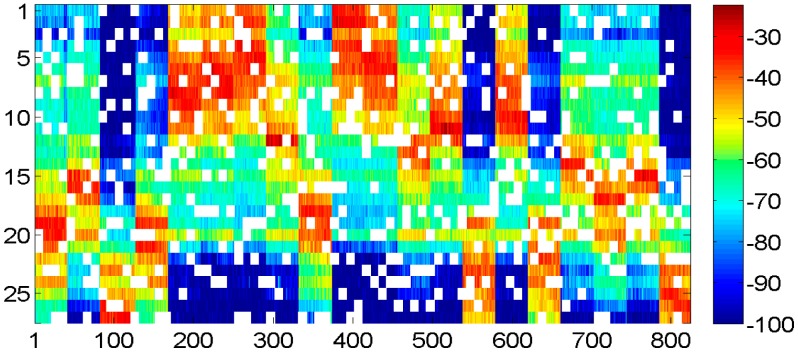
Radio map with five access points are powered off randomly.

In order to simulate the working on-demand strategy in green WLAN, we suppose there are five access points randomly powered off for power conservation when we built the radio map. Figure 10 illustrates some of the RSS readings are unavailable and holes appear in the radio map.

**Figure 11.**
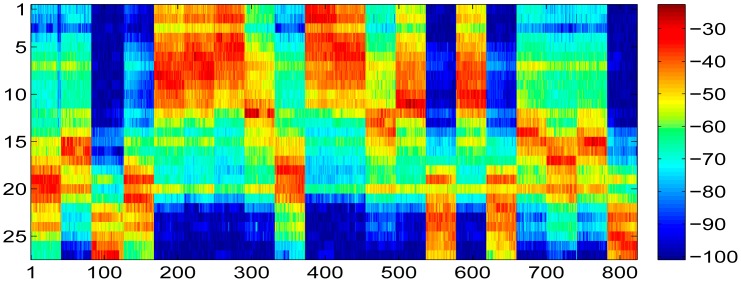
Recovered radio map by SVT when five access points powered off randomly

According to SVT theory, the sensed radio map is subject to low rank and random observation, so that the desired radio map as shown in Figure 9b could be recovered from the one shown in Figure 10 by implementing the shrink iteration. And the recovered result is provided in Figure 11.

**Figure 12.**
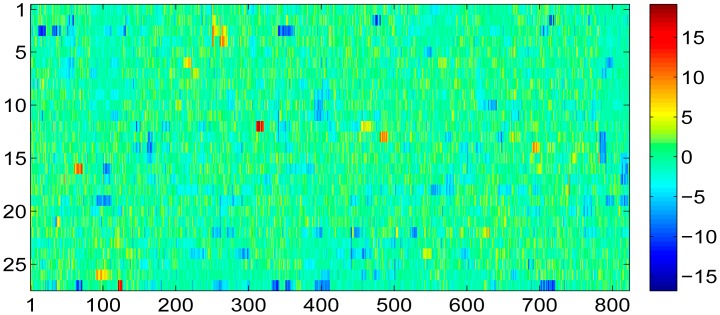
Radio map recovery error between the recovered one and the ideal one.

However, as we stated above, though the SVT theory is able to recover the radio map with holes as precisely as possible, it also introduces recovery errors into the recovered radio map, which is shown in Figure 12. From this figure, we could see that most of the recovery errors introduced to the sensed radio map by SVT theory are around ±5 dBm. For those missing entries, most of the recovery errors are around ±10 dBm.

**Figure 13.**
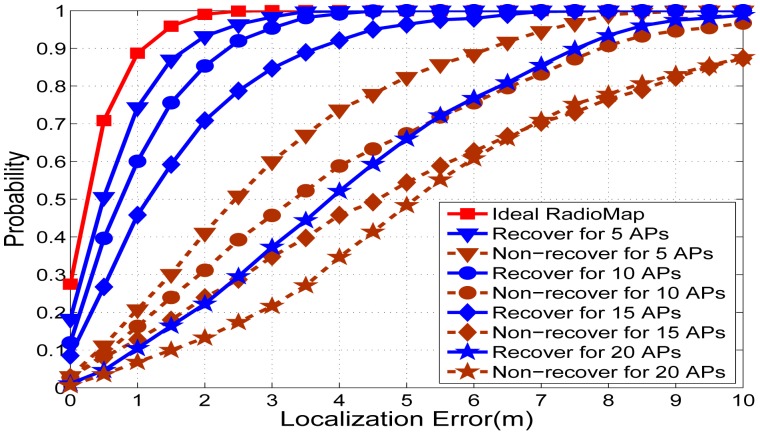
Localization error comparisons for 5, 10, 15 and 20 access points randomly off.

In order to test the proposed system, we simulate three scenarios for comparison. We implement the KNN algorithm based on Equations (1) and (2), and temporally suppose the online RSS readings are free from suffering RSS missing in all these three scenarios. The first scenario is the traditional WLAN, which means all the access points are powered on and available as shown in Figure 9b. The second scenario is the green WLAN and the radio map is recovered by the SVT theory as shown in Figure 11. The third scenario is the missing entries are arbitrarily filled with −100 dBm. Figure 13 provides the details of simulation results for random powering off 5, 10, 15 and 20 access points, respectively, in the green WLAN.

It is concluded that the proposed system makes a very good recovery from the radio map with holes when a small number of the access points are closed, compared with the one filled with a random value. In the meanwhile, Figure 13 also shows that with more access points closing randomly, the RSS recovery performance of SVT decreases. For the 20 powered off access points scenario, the error is unacceptable for indoor localization applications. The number of access points powered off reflects the percentage of the sensed RSS readings from a different perspective. Based on the experimental results, we suggest that the sensed RSS readings in the radio map should not be less than 40%.

**Figure 14.**
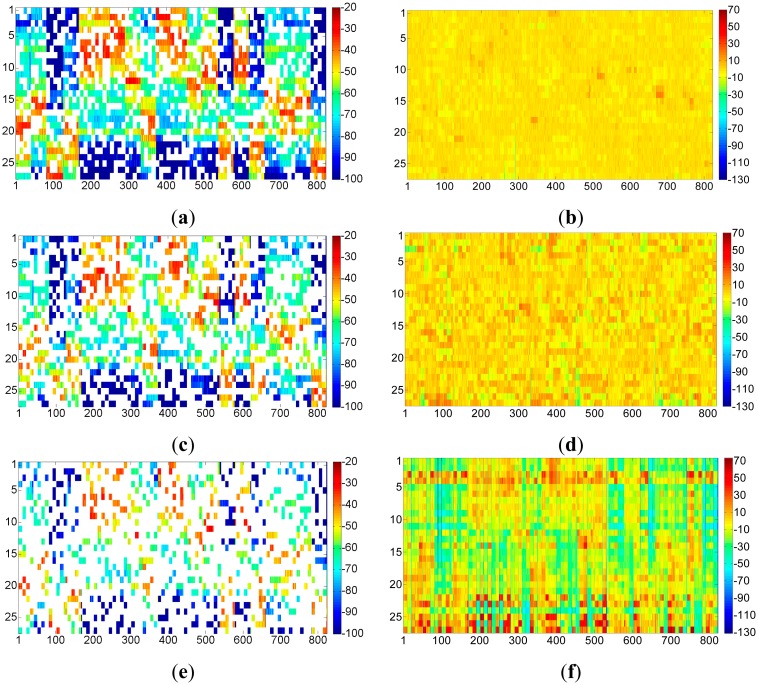
Radio map recovery error in different cases of access points powered off. (**a**) Sensed radio map (10 access points off); (**b**) Recovery Error (10 access points off); (**c**) Sensed radio map (15 access points off); (**d**) Recovery Error (15 access points off); (**e**) Sensed radio map (20 access points off); (**f**) Recovery Error (20 access points off).

In order to overview a much clearer comparisons for radio map recovery by SVT based on Figure 13, we illustrate the radio map recovery results and their recovery error in Figure 14. From these figures, we could predict that with the low data communication requirements in a green WLAN, more access points will be powered off. This will no doubt achieve more energy conservation, however, it will lead to worse performance of the radio map.

**Figure 15.**
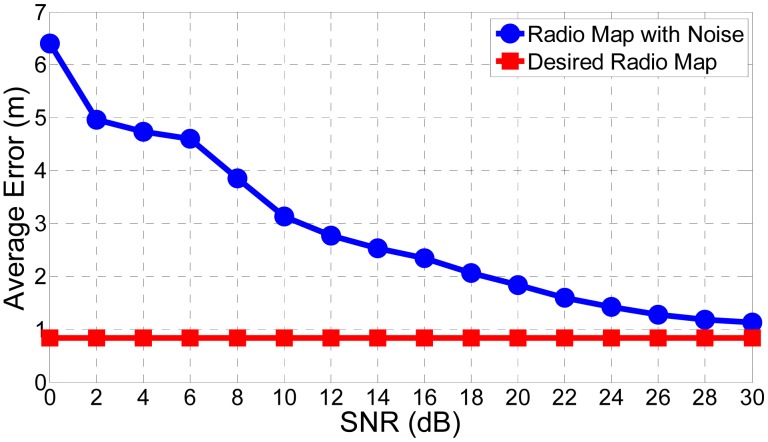
Localization performance affected by radio map.

The recovery error is only one of the factors that will affect the localization performance. Noises are also inevitably important factors. These noise are various, which may be generated from mobile device diversity, complexity of the environment for RSS reading collection, such as temperature, humidity, and signal blocking by humans walking, doors and windows opening, *etc.* Now we will test how the radio map will affect the localization results. We suppose the desired radio map is **Ψ** ∈ ℝ*^m^*^×^*^n^*, and the one with noise is **Ψ̃**∈ ℝ;*^m^*^×^*^n^*. Then we have:

(17) $$\tilde{\Psi }=\Psi +\text{n}$$

where **n** is the noise matrix with zero means representing those noise we discussed above. Figure 15 provides the average localization performance for the desired radio map and the radio map with noise. We suppose there is no hole in the online RSS readings to explain how the radio map built offline affects the results. We could clearly see from this figure that the radio map plays a core role in the localization system. If the radio map is not well built or recovered in the offline phase, the localization performance will be seriously degraded.

### 4.3. Online RSS Reading Recovery

As discussed in Section 3.3, the online RSS readings will also suffer from the missing RSS problem due to the working on-demand strategy used in a green WLAN. Our proposed algorithm will recover it by combining both the online RSS readings and offline RSS readings together, and then recovering it based on a shrink iteration algorithm.

**Figure 16.**
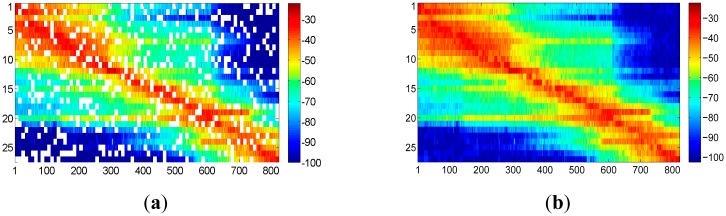
Online RSS reading vector recovery (for 5 access points randomly powered off). (**a**) Sensed online RSS reading vectors; (**b**) Recovered online RSS reading vectors.

Since we recover the radio map, the combined matrix $\Psi_{\text{sensed}}^{\prime }$ will have almost all the entries except the column where the online RSS reading suffers some entries missing. Figure 16a shows the example where 823 RSS readings are collected in the online phase, where we also suppose there are five access points powered off randomly. Then each column is combined with the radio map as shown in Figure 11 and recovered by the SVT theory. After processing 823 times, Figure 16b shows the recovery results.

In the next step, we will test the localization performance with the recovered online RSS readings. The radio map we utilized is the recovered one as shown in Figure 11, which is a more rigorous criterion. The localization method is the KNN algorithm as we discussed in Section 2.1.

**Figure 17.**
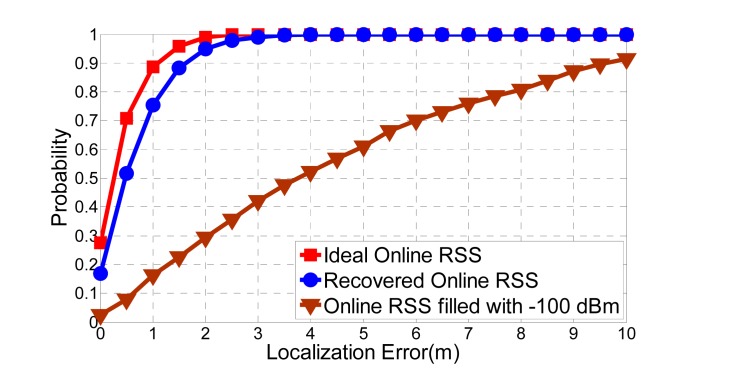
Localization performance for online RSS vectors recovery based on SVT theory.

The localization performance is shown in Figure 17, which is quite good as expected comparing with the results shown in Figure 13. We could see that the recovered online RSS reading vectors outperform those filled with −100 dBm, and its performance is quite close to the ideal one.

## 5. Conclusions

In this paper, we have proposed an RSS-based indoor positioning system in a green WLAN using SVT theory to recover the missing RSS readings for both the offline phase and online phase. A green WLAN employs s working on-demand strategy, where access points will be powered off according to the data communication demands. This strategy would inevitably invalidate the fingerprint algorithm used to estimate the mobile device location due to different RSS readings omission in the offline and online phase. We modeled the missing RSS readings as a convex relaxation of a rank minimization problem. Based on this assumption, we propose to utilize a SVT shrink iteration to recover the radio map with part of the available RSS readings. For the online RSS readings, we propose to combine it together with the radio map, and then utilize the SVT theory for recovery. We tested the proposed system in a typical office environment, and the experiment results shows this could make the recovered radio map achieve an acceptable localization performance, even when 60% (fifteen of twenty seven) access points are unavailable. However, if more access points could not be sensed, the proposed system will introduce large errors into the recovered radio map, which will seriously degrade the localization performance. For the online phase RSS readings recovery, the feasibility and performance are also tested. The proposed system could achieve almost the same performance and outperform the conventional method.
